# Trends in pediatric traumatic brain injury-related mortality in the United States from 1999 to 2024: a retrospective cross-sectional analysis

**DOI:** 10.1097/MS9.0000000000005464

**Published:** 2026-08-06

**Authors:** Asad Ali Ahmed Cheema, Misbah Bibi, Zubia Usman, Abdul Moeez Awais, Zubair Ahmed, Eman Fatima, Fabeha Zafar, Muhammad Noshab

**Affiliations:** a Department of Medicine, International School of Medicine, International University of Kyrgyzstan, Bishkek, Kyrgyzstan; b Department of Medicine, Bahria University Medical and Dental College, Karachi, Pakistan; c Department of Medicine, Punjab Medical College, Faisalabad, Pakistan; d Department of Medicine, Islamic International Medical College, Riphah University, Rawalpindi, Pakistan; e Department of Medicine, Shaikh Khalifa Bin Zayed Al Nahyan Medical and Dental College, Lahore, Pakistan; f Department of Medicine, Foundation University and Medical College, Islamabad, Pakistan; g Department of Medicine, Dow University of Health Sciences, Karachi, Pakistan

**Keywords:** CDC WONDER, joinpoint regression, mortality, pediatric traumatic brain injury, United States

## Abstract

**Background:**

Pediatric traumatic brain injury (pTBI) remains a preventable cause of death among U.S. children and adolescents. Prior national CDC WONDER-based analyses described pTBI mortality through 2017; however, updated surveillance is needed to evaluate post-2017 and post-2020 trends.

**Methods:**

We conducted a population-based repeated cross-sectional analysis using the CDC WONDER Multiple Cause of Death data from 1999 to 2024 among U.S. residents aged 0–19 years with pTBI-related ICD-10 codes recorded anywhere on the death certificate. Crude mortality rates (CMRs) per 1 000 000 population were used to describe the absolute mortality burden. Joinpoint regression estimated the annual percent change (APC) and the average APC (AAPC).

**Results:**

From 1999 to 2024, 128 852 pTBI-related deaths occurred among individuals aged 0–19 years. Overall CMR declined from 89.78 per 1 000 000 population in 1999 to 46.63 per 1 000 000 population in 2024, with significant declines from 1999 to 2006 and from 2006 to 2010, a significant increase from 2013 to 2022, and no statistically significant change from 2022 to 2024. Males accounted for more deaths than females (91 852 vs. 37 000), and adolescents aged 15–19 years accounted for the largest number of deaths (82 409). Non-Hispanic American Indian or Alaska Native children had the highest annual CMRs in both bridged-race and single-race periods. State-level burden remained geographically heterogeneous, with Wyoming the highest and Massachusetts the lowest during 1999–2020, and Montana the highest and Massachusetts the lowest during 2021–2024.

**Conclusion:**

U.S. pTBI-related crude mortality burden declined overall from 1999 to 2024, but recent increases and persistent demographic and geographic differences highlight the need for continued surveillance and targeted prevention.

## Introduction

Pediatric traumatic brain injury (pTBI) remains an important cause of injury-related death and long-term disability among children and adolescents. Survivors may experience persistent cognitive, behavioral, educational, and psychosocial consequences, while fatal pTBI represents a largely preventable public health burden ^[^1–3^]^. This topic is particularly relevant to trauma surgery, neurosurgery, emergency medicine, critical care, and public health because outcomes after severe pediatric head injury may be influenced by prevention, timely recognition, prehospital stabilization, access to pediatric trauma and neurosurgical services, and definitive critical care. The mortality burden is also unevenly distributed and may differ substantially by age, sex, race and ethnicity, geographic region, urban-rural status, and state-level context^[^4–7^]^.

HIGHLIGHTS

- From 1999 to 2024, 128 852 pTBI-related deaths occurred among U.S. children and adolescents.
- Overall, CMR declined from 89.78 to 46.63 per 1 000 000 population, but it increased significantly from 2013 to 2022.
- The mortality burden was greatest among males and adolescents aged 15–19 years, who accounted for 91 852 and 82 409 deaths, respectively.
- The geographic burden was heterogeneous, with Wyoming having the highest burden during 1999–2020, Montana the highest during 2021–2024, and Massachusetts the lowest in both periods.

Prior national studies have characterized pTBI-related mortality among U.S. children and adolescents, including a CDC WONDER-based analysis by Cheng *et al* covering 1999–2017^[^7^]^. That study demonstrated substantial mortality declines through 2013 followed by an increase through 2017 and identified important differences by sex, age, urbanicity, state, injury mechanism, and intent. However, several clinically and epidemiologically important questions remain insufficiently characterized. It remains unclear whether the post-2013 increase persisted, stabilized, or changed after 2017; whether previously observed national patterns changed during the COVID-19 era and subsequent years; and which demographic and geographic groups continue to experience the greatest contemporary burden. In addition, the transition from bridged-race to single-race mortality reporting complicates interpretation of long-term racial and ethnic trends, while updated analyses are needed to characterize recent state-level and metropolitan–nonmetropolitan differences. The extent to which recent firearm-related mortality increases reflect homicide, suicide, or unintentional discharge also has distinct implications for prevention policy and clinical risk-reduction strategies.

To address these gaps, we conducted a population-based repeated cross-sectional analysis of CDC WONDER Multiple Cause of Death data for U.S. residents aged 0–19 years. We evaluated national pTBI-related mortality trends from 1999 through 2024 using crude mortality rates (CMRs) and Joinpoint regression, characterized differences by sex, age, race and ethnicity, region, state, urban-rural status, and place of death, and examined available injury mechanism–specific patterns and firearm mortality patterns by intent. By extending national surveillance through 2024, separating bridged-race and single-race reporting periods, and integrating contemporary demographic, geographic, mechanism-specific, and intent-specific analyses, this study provides an updated assessment of where the pTBI-related mortality burden remains concentrated. These findings are intended to inform clinicians, trauma systems, neurosurgical services, policymakers, and injury-prevention programs regarding populations and geographic areas in which targeted surveillance, prevention, and systems-level planning may be most needed.

## Methodology

### Study design, data source, and population

We conducted a population-based, repeated cross-sectional analysis of pTBI-related mortality in the United States from 1 January 1999 through 31 December 2024. Mortality data were obtained from the Centers for Disease Control and Prevention Wide-ranging Online Data for Epidemiologic Research (CDC WONDER) Multiple Cause of Death database. This database contains national mortality and population data derived from death certificates for U.S. residents and includes demographic information, one underlying cause of death, and multiple contributing causes of death, all coded according to the International Classification of Diseases, Tenth Revision (ICD-10)^[^8^]^. Because 1999 was the first year in which U.S. mortality records in CDC WONDER were classified under ICD-10, restricting the analysis to 1999–2024 ensured the use of a single ICD revision throughout the study period and avoided the coding discontinuity that could arise from combining or crosswalking mortality data coded under ICD-9 and ICD-10.

The analytic population included deaths among U.S. residents aged 0–19 years in which at least one pre-specified pTBI-related ICD-10 code was recorded anywhere on the death certificate, either as the underlying cause or as a contributing cause of death. Records were excluded if the decedent was older than 19 years, the death occurred outside the study period, or no pre-specified pTBI-related ICD-10 code was present in the multiple-cause-of-death record. Nonfatal traumatic brain injuries, emergency department visits, hospitalizations, and deaths without a pTBI-related ICD-10 code were not included. Because this analysis used de-identified, publicly available aggregate mortality data, no individual-level patient information was accessed.

### Case definition and ICD-10 code selection

pTBI-related deaths were identified using a prespecified ICD-10 multiple-cause-of-death code set selected before data extraction. The code set was based on established fatal TBI mortality surveillance definitions and prior CDC WONDER-based TBI mortality analyses^[^7,9–11^]^. Codes were selected to capture traumatic injuries involving the head, skull, cranial nerves, and brain, as well as sequelae of traumatic head injury. The included ICD-10 codes were S01.0–S01.5, S01.7–S01.9, S02.0, S02.1, S02.3, S02.7–S02.9, S04.0, S06.0–S06.9, S07.0, S07.1, S07.8, S07.9, S09.7–S09.9, T90.1, T90.2, T90.4, T90.5, T90.8, and T90.9.

The multiple-cause-of-death field was used because pTBI may contribute to death in combination with other injuries, complications, or external causes and may not always be coded as the single underlying cause of death. Restricting case identification to the underlying cause of death alone could therefore underestimate pTBI-related mortality. Accordingly, a death was classified as pTBI-related if any prespecified pTBI ICD-10 code appeared anywhere in the death-certificate record. External cause-of-injury codes were not required for inclusion in the case definition. The complete CDC WONDER query criteria are provided in Supplemental Digital Content Table S1, available at https://links.lww.com/MS9/B340 to improve reproducibility.

### Variables and subgroup definitions

Mortality data were extracted by calendar year, age group, sex, race and ethnicity, U.S. census region, state, urban-rural status, and place of death. Age was categorized as younger than 1 year, 1–4 years, 5–9 years, 10–14 years, and 15–19 years. Race and ethnicity were analyzed according to the CDC WONDER reporting structure. Bridged-race categories were analyzed for 1999–2017, and single-race categories were analyzed separately for 2018–2024 to avoid mixing race classification systems. Race and ethnicity categories included children categorized as non-Hispanic White, non-Hispanic Black or African American, non-Hispanic American Indian or Alaska Native, Hispanic or Latino, and non-Hispanic Asian or Pacific Islander.

U.S. census regions were classified as Northeast, Midwest, South, and West according to U.S. Census Bureau definitions^[^12^]^. Urban–rural status was classified as metropolitan or nonmetropolitan using the 2013 National Center for Health Statistics Urban–Rural Classification Scheme for Counties^[^13^]^. Urban–rural data were available through 2021 in the extracted dataset; therefore, urban-rural analyses were limited to 1999–2021 and were not interpreted as covering the full 1999–2024 study period. Place of death was categorized as inpatient medical facility, outpatient or emergency department, dead on arrival, decedent’s home, hospice facility, nursing home or long-term care facility, other location, or unknown location. State-level results were summarized separately for 1999–2020 and 2021–2024 to present both long-term and contemporary geographic burden without combining periods of unequal duration.

Data extraction was performed independently by two authors using a prespecified CDC WONDER query strategy that applied the same age restriction, study period, ICD-10 code list, and subgroup definitions across analyses. Extracted death counts, population denominators, CMRs, confidence intervals, and Joinpoint input files were cross-checked. Any discrepancies were resolved by re-review of the original CDC WONDER outputs by a third author. Suppressed or unavailable CDC WONDER outputs were not imputed.

An additional injury-mechanism–specific analysis was conducted for 1999–2020, the period for which the extracted CDC WONDER mechanism data were available. The evaluated categories were falls, firearms, motor vehicle traffic, other pedestrian injuries, other land-transport injuries, other specified classifiable injuries, other specified injuries not elsewhere classified, injuries involving being struck by or against an object, and unspecified injuries. Annual CMRs, Joinpoint-derived annual percent changes (APCs), and average APCs (AAPCs) were analyzed descriptively.

To further characterize the post-2013 increase in firearm-related mortality, we conducted a secondary intent-stratified analysis for 1999–2020, corresponding to the period covered by the injury-mechanism-specific analysis. The same multiple-cause-of-death pTBI case definition was retained, while firearm-related deaths were classified according to the ICD-10 external-cause code recorded as the underlying cause of death: unintentional firearm discharge (W32–W34), intentional self-harm by firearm (X72–X74), assault by firearm (X93–X95), firearm discharge of undetermined intent (Y22–Y24), and legal intervention involving firearm discharge (Y35.0). Annual death counts and CMRs per 1 000 000 population were calculated separately for each intent category. Intent-specific Joinpoint regression was performed for categories with sufficient annual observations to support stable trend estimation. Suppressed or unreliable estimates were not imputed, and categories with insufficient reportable observations were summarized descriptively.

### Mortality rate calculation

The primary mortality measure was the CMR per 1 000 000 population. CMRs were calculated by dividing the number of pTBI-related deaths by the corresponding population denominator and multiplying by 1 000 000. Crude rates and corresponding 95% confidence intervals were extracted from CDC WONDER for the overall population and for prespecified demographic, geographic, and place-of-death categories, as well as for available subgroup analyses.

CMRs were selected to describe the absolute population-level burden of pTBI-related mortality within the restricted pediatric population aged 0–19 years. Age-adjusted rates were not used as the primary mortality measure because the CDC WONDER standard age-adjusted rates are not generated for the selected 5-year pediatric age groups used in this analysis. Therefore, age was addressed through prespecified age-specific analyses using the following strata: younger than 1 year, 1–4 years, 5–9 years, 10–14 years, and 15–19 years. Comparisons across sex, race and ethnicity, geographic, urban-rural, and other subgroups were interpreted as descriptive comparisons of crude mortality burden rather than as age-standardized comparisons of mortality risk.

### Joinpoint regression analysis

Temporal trends in pTBI-related mortality were evaluated using the National Cancer Institute Joinpoint Regression Program, version 5.3.0. Calendar year was modeled as the independent variable, and the natural logarithm of the annual CMR was used as the dependent variable. For the complete 1999–2024 annual time series, models were permitted to contain between zero and four joinpoints. Candidate joinpoint locations were evaluated using the Grid Search method, and the final number of joinpoints was selected using the Monte Carlo permutation-test procedure implemented in the Joinpoint Regression Program. The simplest model was retained unless the addition of one or more joinpoints resulted in a statistically significant improvement in model fit. The Bayesian Information Criterion was not used for model selection.

APC and AAPC were calculated with corresponding 95% confidence intervals using the parametric method. All *P*-values were two-sided and reported to three decimal places; values below 0.001 were reported as *P* < 0.001. Statistical significance was defined as *P* < 0.05. Segments with *P*-values greater than or equal to 0.05 were interpreted as showing no statistically significant change. For analyses covering shorter periods or containing suppressed or unavailable annual estimates, the maximum number of joinpoints was constrained by the number of eligible annual observations and the software’s minimum-observation requirements. No Joinpoint model was fitted when the available time series was insufficient for stable segmented regression.

### Bias, confounding, and interpretive framework

As CDC WONDER provides aggregate death-certificate data rather than individual-level clinical records, multivariable adjustment for potential confounders was not possible. Potential confounders that could not be directly measured included injury severity, intent of injury, polytrauma, prehospital response time, transport distance, trauma-center access, neurosurgical availability, socioeconomic status, insurance status, comorbid conditions, restraint use, firearm access or storage practices, and local injury-prevention policies. Death-certificate data may also be affected by variations in certifier documentation, medical examiner involvement, coding practices, and the completeness of information on injury circumstances.

Therefore, subgroup comparisons were interpreted descriptively as differences in crude population-level mortality burden rather than as adjusted estimates of individual-level risk. Similarly, observed temporal changes were interpreted as ecological surveillance trends rather than as causal effects of specific policies, clinical interventions, injury mechanisms, or changes in health-care delivery.

### Ethical considerations and reporting guidelines

This study used de-identified, publicly available mortality data from CDC WONDER and did not involve direct interaction with human participants or access to identifiable private information. Therefore, institutional review board approval and informed consent were not required. The study was reported in accordance with the Strengthening the Reporting of Observational Studies in Epidemiology (STROBE) guideline^[^14^]^ and the Strengthening the Reporting of Cohort, Cross-sectional, and Case-control Studies in Surgery (STROCSS) 2024 guideline, where applicable^[^15^]^. Reporting of artificial intelligence use in this study adhered to the Transparency in the Reporting of Artificial Intelligence (TITAN) guideline, and the completed TITAN checklist is provided in the Supplementary Material^[^16^]^.

## Results

Between 1999 and 2024, 128 852 pTBI-related deaths occurred among individuals aged 0–19 years in the United States. All mortality rates are reported as CMRs per 1 000 000 population and should be interpreted as descriptive estimates of absolute mortality burden rather than age-standardized mortality risk. Among recorded place-of-death categories, deaths occurred most commonly in other locations (36.17%), inpatient medical facilities (26.30%), outpatient or emergency facilities (19.28%), and at home (14.38%) (Supplemental Digital Content Table S2, available at: https://links.lww.com/MS9/B340).

### Overall temporal trends

**Figure 1.**
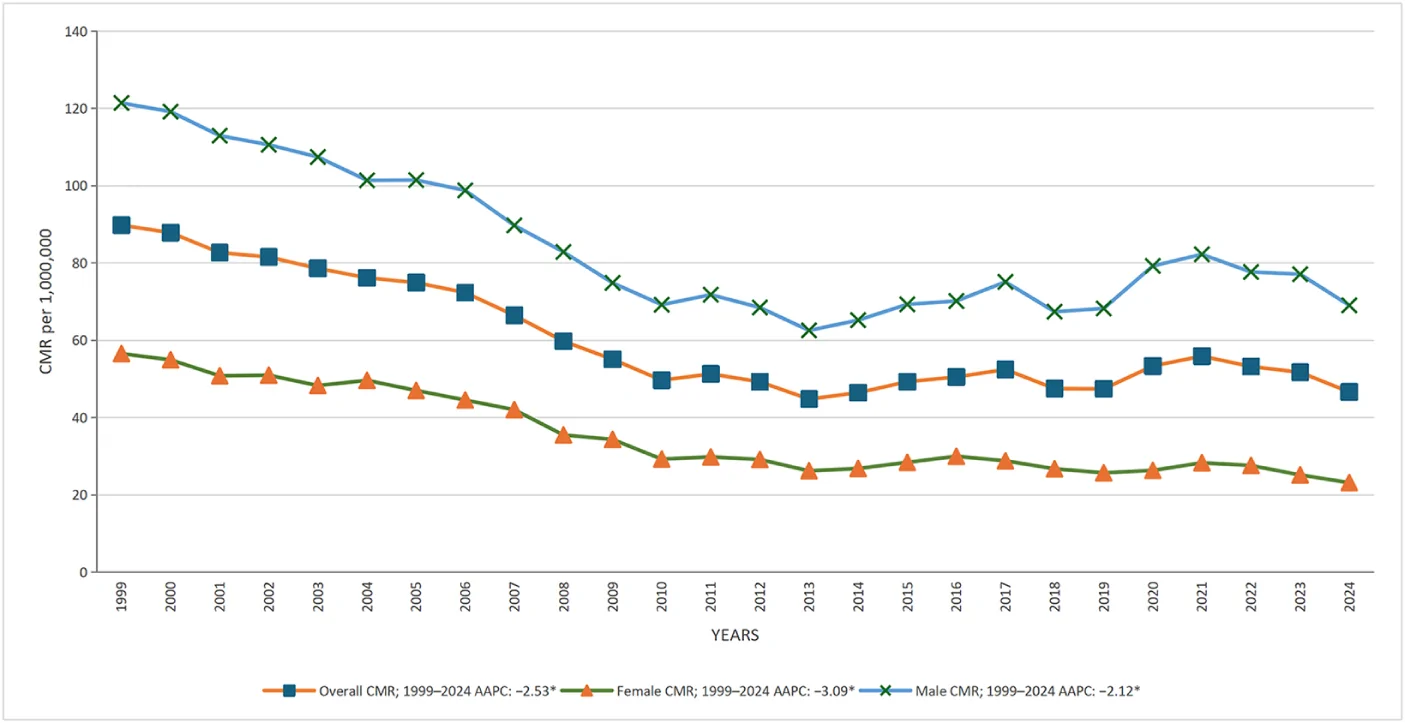
Overall and sex-stratified pTBI-related CMRs in the United States, 1999–2024. Annual CMRs were consistently higher among males than among females.

CMR, crude mortality rate; pTBI, pediatric traumatic brain injury. CMRs are reported per 1 000 000 population.

The overall CMR declined from 89.78 per 1 000 000 population in 1999 to 46.63 per 1 000 000 in 2024. Joinpoint regression showed significant declines from 1999 to 2006 (APC: −3.07%; 95% CI: −4.31 to −1.81; *P* < 0.001) and from 2006 to 2010 (APC: −8.32%; 95% CI: −13.26 to −3.09; *P* = 0.005). The trend showed no statistically significant change from 2010 to 2013, increased significantly from 2013 to 2022 (APC: 1.70%; 95% CI: 0.37 to 3.05; *P* = 0.017), and showed no statistically significant change from 2022 to 2024. Across the full study period, the overall AAPC declined significantly (AAPC: −2.53%; 95% CI: −4.32 to −0.70; *P* = 0.007) (Fig. 1; Supplemental Digital Content Tables S3 and S4, available at: https://links.lww.com/MS9/B340).

### Sex- and age-specific trends

Males accounted for more pTBI-related deaths than females (91 852 vs. 37 000), and annual CMRs were consistently higher among males throughout the study period. From 1999 to 2024, the CMR declined from 121.39 to 69.03 among males and from 56.54 to 23.17 among females. Overall mortality trends declined significantly in both males (AAPC: −2.12%; 95% CI: −3.25 to −0.97; *P* < 0.001) and females (AAPC: −3.09%; 95% CI: −4.18 to −1.99; *P* < 0.001). However, males had a significant increase from 2013 to 2021, followed by no statistically significant change from 2021 to 2024 (Fig. 1; Supplemental Digital Content Tables S4 and S5, available at: https://links.lww.com/MS9/B340).

**Figure 2.**
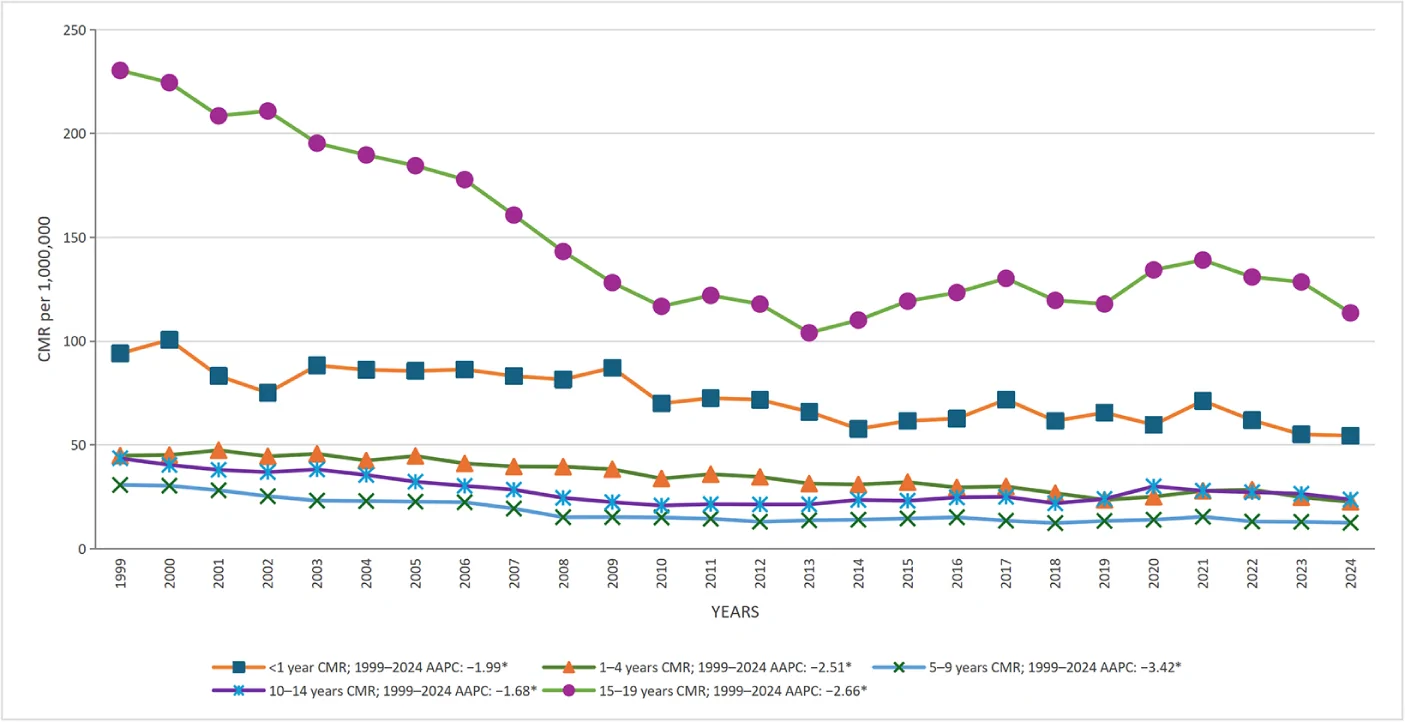
Age-specific pTBI-related CMRs in the United States, 1999–2024. Adolescents aged 15–19 years had the highest annual CMRs throughout the study period.

CMR, crude mortality rate; pTBI, pediatric traumatic brain injury. CMRs are reported per 1 000 000 population.

Age-stratified analyses showed that adolescents aged 15–19 years accounted for the largest number of deaths (82 409), followed by children aged 10–14 years (15 308), children aged 1–4 years (14 325), children aged 5–9 years (9317), and infants younger than 1 year (7493). Annual CMRs declined from 1999 to 2024 in every age group, but adolescents aged 15–19 years consistently had the highest annual CMRs. Across 1999-2024, the AAPC declined significantly in all five age groups. However, segmented analyses showed that CMRs among children aged 10–14 years increased significantly from 2010 to 2024, while CMRs among adolescents aged 15–19 years increased significantly from 2013 to 2021 before showing no statistically significant change from 2021 to 2024 (Fig. 2; Supplemental Digital Content Tables S4 and S6, available at: https://links.lww.com/MS9/B340).

### Racial and ethnic patterns

**Figure 3.**
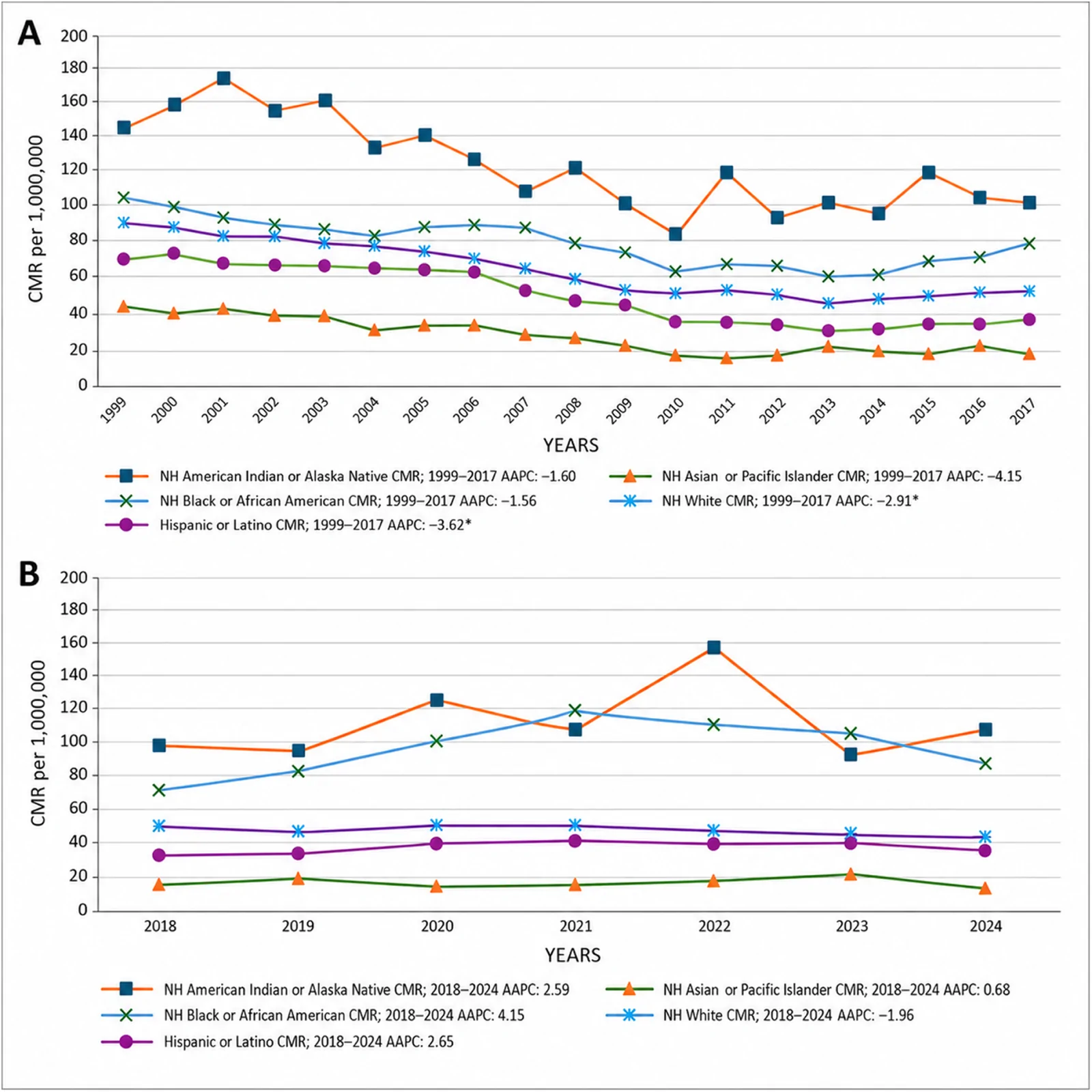
Race- and ethnicity-stratified pTBI-related CMRs in the United States. Panel A shows bridged-race and ethnicity categories from 1999 to 2017; Panel B shows single-race and ethnicity categories from 2018 to 2024.

CMR, crude mortality rate; pTBI, pediatric traumatic brain injury. CMRs are reported per 1 000 000 population. Bridged-race and single-race estimates should be interpreted separately.

Racial and ethnic findings were interpreted separately for bridged-race and single-race periods to avoid mixing race classification systems. During the bridged-race period from 1999 to 2017, annual CMRs were highest among non-Hispanic American Indian or Alaska Native children at both the beginning and end of the period, decreasing from 145.60 in 1999 to 102.83 in 2017. Over the same period, annual CMRs declined among non-Hispanic Black or African American, non-Hispanic White, Hispanic or Latino, and non-Hispanic Asian or Pacific Islander children. Overall AAPCs declined significantly among non-Hispanic White and Hispanic or Latino children, whereas no statistically significant overall change was observed among non-Hispanic American Indian or Alaska Native, non-Hispanic Asian or Pacific Islander, or non-Hispanic Black or African American children. Notably, non-Hispanic Black or African American children had a significant increase from 2013 to 2017 (Fig. 3, Panel A; Supplemental Digital Content Tables S4 and S7, available at: https://links.lww.com/MS9/B340).

During the single-race period from 2018 to 2024, annual CMRs remained highest among non-Hispanic American Indian or Alaska Native children, increasing from 97.17 in 2018 to 108.26 in 2024. Non-Hispanic Black or African American children had a significant increase from 2018 to 2021 (APC: 18.83%; 95% CI: 3.03% to 37.04%; *P* = 0.035), followed by no statistically significant change from 2021 to 2024. Other single-race and ethnicity groups showed no statistically significant change during 2018–2024 (Fig. 3, Panel B; Supplemental Digital Content Tables S4 and S8, available at: https://links.lww.com/MS9/B340).

### Geographic and urban–rural patterns

**Figure 4.**
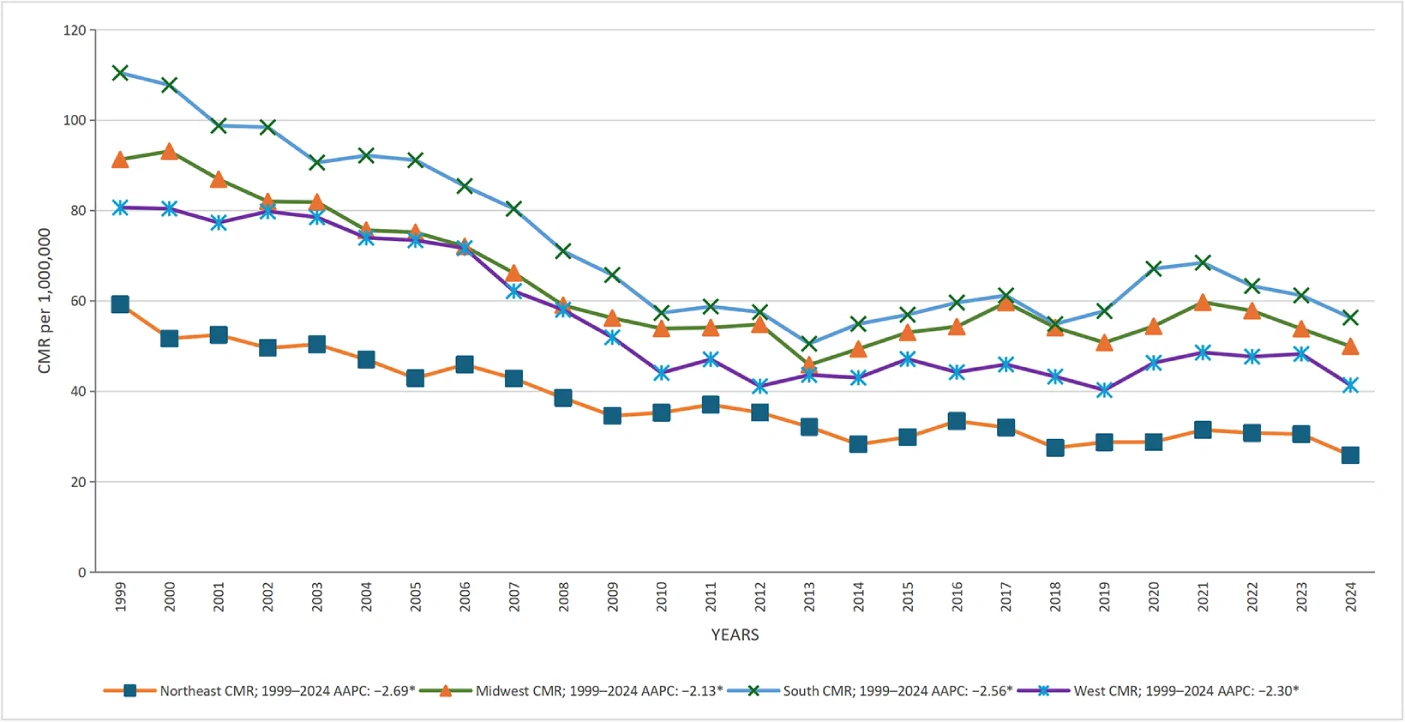
Region-stratified pTBI-related CMRs in the United States, 1999–2024. Annual CMRs are shown by U.S. Census region.

CMR, crude mortality rate; pTBI, pediatric traumatic brain injury. CMRs are reported per 1 000 000 population.

Geographic variation persisted throughout the study period. The South accounted for the largest number of pTBI-related deaths (57 286), followed by the Midwest (29 458), the West (28 487), and the Northeast (13 621). Annual CMRs declined from 1999 to 2024 in all four regions. Joinpoint analysis showed significant overall declines across all regions, although rates in the South increased significantly from 2013 to 2021 and then showed no statistically significant change from 2021 to 2024 (Fig. 4; Supplemental Digital Content Tables S4 and S9, available at: https://links.lww.com/MS9/B340).

**Figure 5.**
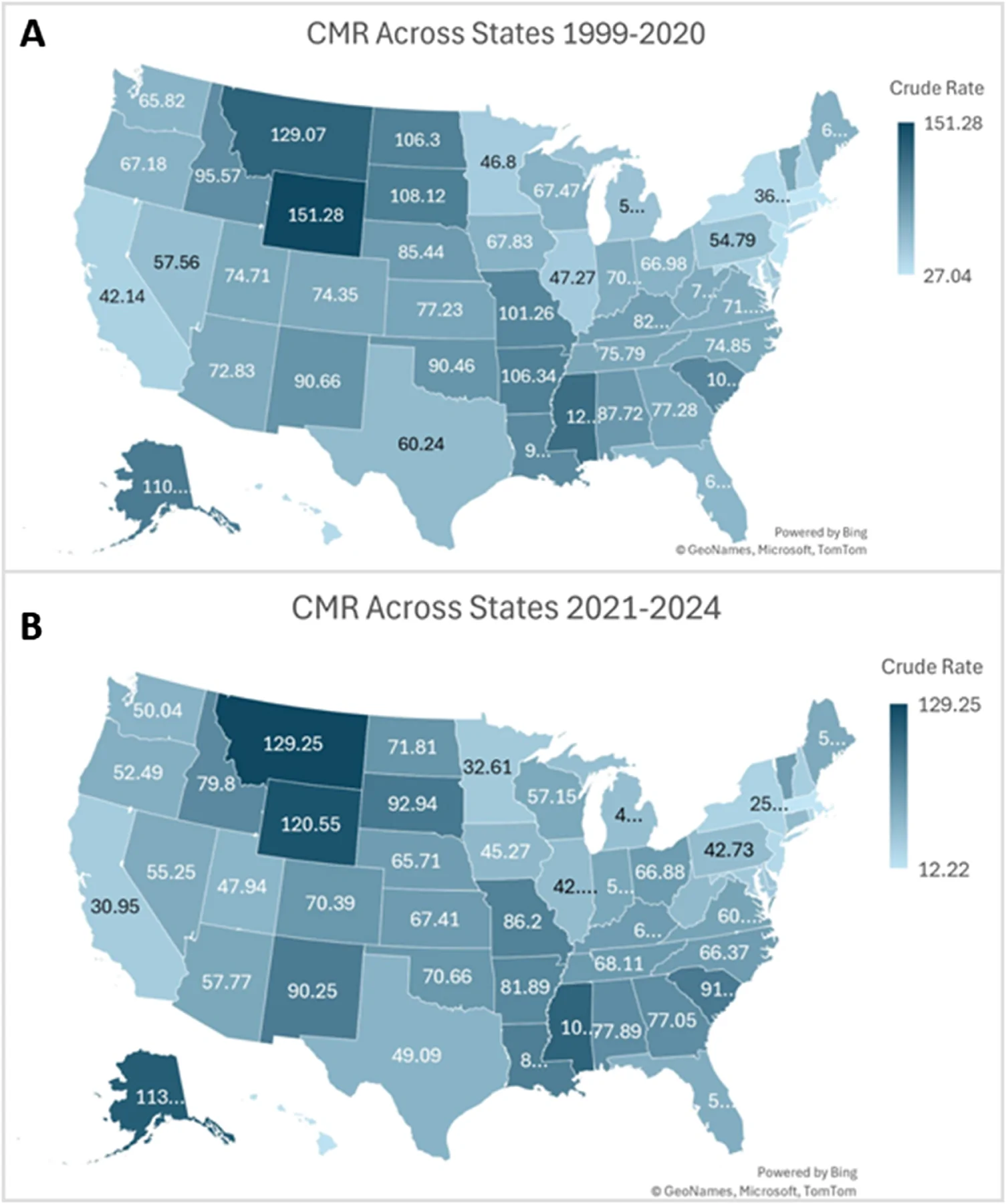
State-level pTBI-related CMRs in the United States. Panel A shows state-level CMRs from 1999 to 2020; Panel B shows state-level CMRs from 2021 to 2024.

CMR, crude mortality rate; pTBI, pediatric traumatic brain injury. CMRs are reported per 1 000 000 population. Panel B represents a shorter contemporary interval and should not be interpreted as a long-term state-specific trend.

State-level burden was summarized separately for 1999–2020 and 2021–2024. From 1999 to 2020, Wyoming had the highest state-level CMR (151.28), whereas Massachusetts had the lowest (27.04). From 2021 to 2024, Montana had the highest state-level CMR (129.25), whereas Massachusetts again had the lowest (12.22) (Fig. 5, Panels A and B; Supplemental Digital Content Tables S10 and S11, available at: https://links.lww.com/MS9/B340).

**Figure 6.**
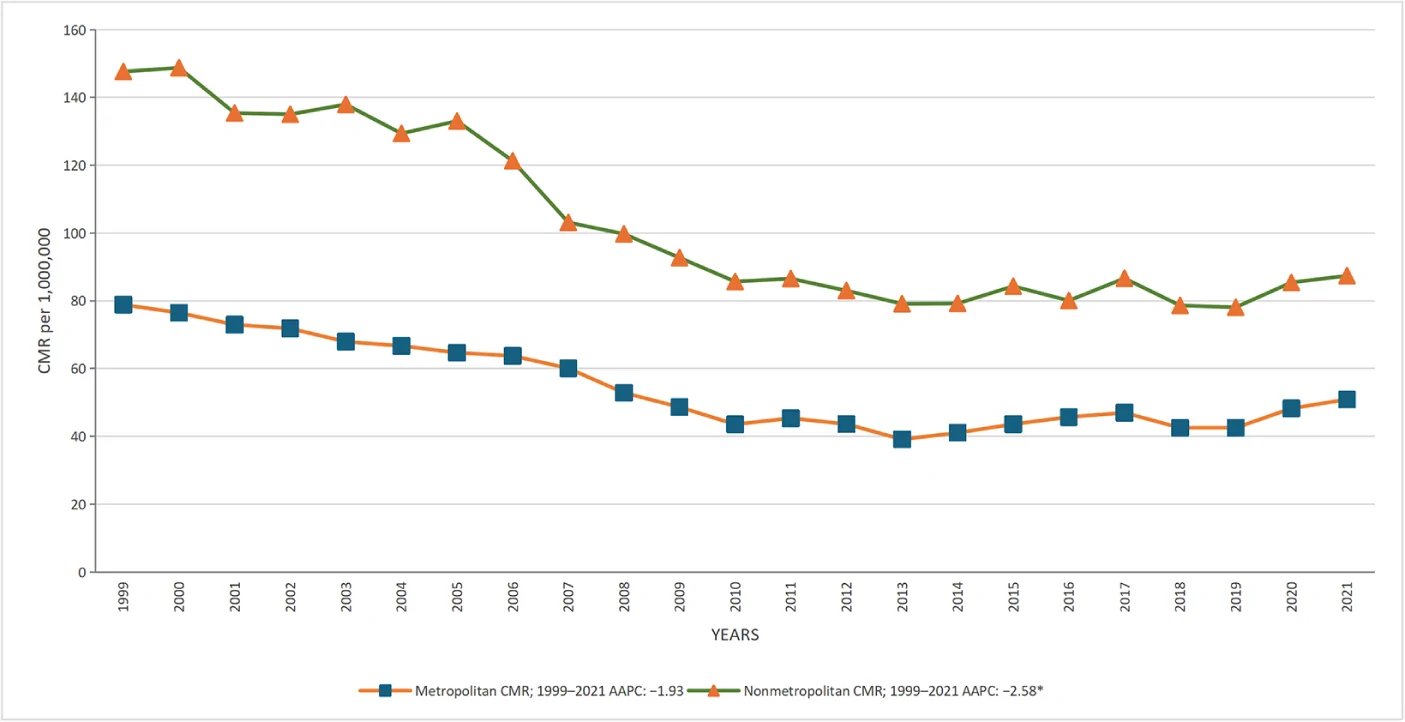
Urban-rural pTBI-related CMRs in the United States, 1999–2021. Urban–rural analyses were available through 2021.

CMR, crude mortality rate; pTBI, pediatric traumatic brain injury. CMRs are reported per 1 000 000 population.

Urban-rural analyses were available through 2021. Annual CMRs were higher in nonmetropolitan areas than in metropolitan areas throughout the available period. From 1999 to 2021, the CMR declined from 147.63 to 87.41 in nonmetropolitan areas and from 78.86 to 50.92 in metropolitan areas. Across the entire available period, nonmetropolitan areas had a significant overall decline (AAPC: −2.58%; 95% CI: −3.53 to −1.63; *P* < 0.001), whereas metropolitan areas showed no statistically significant overall change (AAPC: −1.93%; 95% CI: −3.90 to 0.09; *P* = 0.060) (Fig. 6, Supplemental Digital Content Tables S4 and S12, available at: https://links.lww.com/MS9/B340).

### Injury-mechanism–specific patterns, 1999–2020

Motor vehicle traffic had the highest CMR in 1999, at 49.00 per 1 000 000 population (95% CI: 47.47 to 50.53), and declined to 15.93 in 2020 (95% CI: 15.06 to 16.79). This represented a significant overall decline across the available period (AAPC: −5.64%; 95% CI: −6.93 to −4.33). In contrast, the firearm-related CMR increased significantly from 2013 to 2020 (APC: 7.30%; 95% CI: 5.30 to 9.34), reaching 28.66 per 1 000 000 population in 2020 (95% CI: 27.50 to 29.83), although its overall 1999–2020 AAPC was not statistically significant (AAPC: 0.83%; 95% CI: −0.42 to 2.09). Significant overall declines were also observed for falls, other pedestrian injuries, other land-transport injuries, other specified classifiable injuries, other specified injuries not elsewhere classified, injuries involving being struck by or against an object, and unspecified injuries. Detailed annual estimates and Joinpoint results are provided in Supplemental Digital Content Tables S13–S15, available at: https://links.lww.com/MS9/B340.

Intent-stratified analyses indicated that the post-2013 increase in firearm-related pTBI mortality was driven predominantly by intentional injuries rather than unintentional firearm discharges. From 2013 to 2020, the homicide-related firearm CMR increased from 5.78 per 1 000 000 population (95% CI: 5.26 to 6.29) to 11.54 (95% CI: 10.81 to 12.28), while the suicide-related firearm CMR increased from 9.51 (95% CI: 8.84 to 10.17) to 14.97 (95% CI: 14.12 to 15.81). Together, homicide- and suicide-related deaths accounted for approximately 94% of the absolute increase in the overall firearm-related CMR between 2013 and 2020. Homicide-related mortality increased significantly from 2013 to 2018 (APC: 4.74%; 95% CI: 0.64 to 9.00; *P* = 0.028) and increased more rapidly from 2018 to 2020 (APC: 19.69%; 95% CI: 7.51 to 33.25; *P* = 0.005). Suicide-related mortality increased significantly from 2007 to 2020 (APC: 5.85%; 95% CI: 4.73 to 6.99; *P* < 0.001). In contrast, the unintentional firearm-discharge CMR changed from 1.02 in 2013 (95% CI: 0.81 to 1.26) to 1.27 in 2020 (95% CI: 1.02 to 1.51), with no statistically significant change from 2009 to 2020 (APC: 2.17%; 95% CI: −0.30 to 4.71; *P* = 0.082). The CMR for firearm deaths of undetermined intent increased from 0.32 in 2013 (95% CI: 0.21 to 0.46) to 0.85 in 2020 (95% CI: 0.66 to 1.07), whereas legal-intervention estimates were predominantly suppressed and did not support reliable trend analysis. Detailed annual intent-specific estimates and Joinpoint results are provided in Supplemental Digital Content Tables S16–S18, available at: https://links.lww.com/MS9/B340.

## Discussion

In this 26-year national analysis of pTBI-related mortality among U.S. children and adolescents, we identified several important findings. First, crude pTBI-related mortality burden declined substantially from 1999 to 2010, showed no statistically significant change from 2010 to 2013, significantly increased from 2013 to 2022, and then showed no statistically significant change from 2022 to 2024. Second, mortality burden remained unevenly distributed by sex and age, with males accounting for more deaths than females and adolescents aged 15–19 years accounting for the largest number of deaths. Third, racial/ethnic and geographic differences persisted, including high annual CMRs among non-Hispanic American Indian or Alaska Native children and substantial state-level variation across both the 1999–2020 and 2021–2024 periods. Because all rates were crude rather than age-adjusted, these subgroup patterns should be interpreted as descriptive differences in absolute mortality burden rather than age-standardized comparisons of mortality risk.

The literature used to contextualize these findings includes national mortality-surveillance analyses, administrative cohorts, cross-sectional surveys, program evaluations, reviews, meta-analyses, clinical guidelines, and policy documents. Because these sources address different populations, exposures, and outcomes, no single evidence-grading framework was applied. National surveillance and administrative studies were interpreted primarily as descriptive or associational evidence, whereas single-center, ecological, and program-evaluation studies were considered contextual or hypothesis-generating. Reviews, meta-analyses, and evidence-informed guidelines were used as syntheses for specific clinical or public-health questions.

The early decline in pTBI-related mortality is consistent with prior population-based mortality-surveillance and administrative analyses, including the national CDC WONDER study by Cheng *et al*, which documented declining mortality through 2013 followed by an increase through 2017^[^3,7,9,10,17,18^]^. These studies provide broad population coverage but cannot establish why the trends changed. Evidence relevant to prevention is also predominantly observational and includes professional child-passenger-safety recommendations, restraint-use studies, traffic-safety program evaluations, a meta-analysis of graduated-driver-licensing laws, a single-center severe-pTBI protocol-implementation study, and a national cross-sectional assessment of emergency department pediatric readiness^[^19–26^]^. Additional observational studies, national surveys, and systematic reviews describe adolescent driving risks, firearm-related injury, substance-related risks, and sports-related concussion^[^27–35^]^. Collectively, this evidence supports plausible roles for motor-vehicle safety, pediatric emergency readiness, standardized acute care, and mechanism-specific prevention, but it does not demonstrate that any individual policy or program caused the mortality trends observed here. In light of the increase from 2013 to 2022 and the intent-specific firearm findings, prevention priorities should remain directly aligned with the populations and mechanisms showing persistent burden, including adolescent road safety, suicide prevention, community-violence reduction, secure firearm storage, and timely access to pediatric trauma care.

The intent-specific analysis clarified that the post-2013 increase in firearm-related pTBI mortality was driven overwhelmingly by intentional injuries rather than unintentional firearm discharges. Both homicide- and suicide-related firearm mortality increased substantially, together accounting for approximately 94% of the observed increase in the overall firearm-related CMR from 2013 to 2020. Homicide-related mortality increased particularly rapidly during 2018–2020, whereas suicide-related mortality showed a sustained significant increase from 2007 to 2020. These findings have important prevention implications because the relevant strategies differ by injury intent. Homicide-related mortality requires approaches addressing interpersonal and community violence and access to firearms among populations at elevated risk. Suicide-related mortality requires timely identification of suicide risk, access to behavioral-health and crisis services, and lethal-means safety. In contrast, prevention of unintentional firearm discharge more directly emphasizes secure firearm storage, caregiver education, and prevention of unauthorized access by children. These findings remain descriptive and should not be interpreted as evidence that any specific policy, program, or exposure caused the observed intent-specific trends.

Age-specific findings have important clinical implications. Adolescents aged 15–19 years accounted for the largest number of pTBI-related deaths and consistently had the highest annual CMRs, whereas infants younger than 1 year represented a smaller absolute number of deaths but remain a clinically vulnerable group. These groups require different prevention frameworks. Adolescent prevention is more closely aligned with reducing risk exposure in transportation, violence, sports, and other high-risk environments. In contrast, infant pTBI prevention depends more heavily on caregiver education, safe home environments, recognition of injury warning signs, and prevention of abusive head trauma. Evidence concerning abusive head trauma and infant injury patterns is derived primarily from retrospective administrative database analyses, clinical observational studies, narrative reviews, and caregiver-education literature rather than from randomized prevention trials^[^36–41^]^. These sources consistently identify infant vulnerability and relevant injury circumstances but provide more limited evidence regarding the comparative effectiveness of specific prevention programs. Because access to traditional caregiver education may be limited in rural and under-resourced settings, digital health tools may complement established clinician-led counseling and abusive head trauma prevention programs by extending the reach of educational materials and facilitating adaptation to different literacy, language, and informational needs. The cited artificial intelligence literature is a bibliometric mapping study that identifies emerging themes in patient education rather than an intervention trial that demonstrates improved clinical or prevention outcomes^[^42^]^. Its findings should therefore be interpreted as forward-looking and hypothesis-generating. Artificial intelligence-supported tools require rigorous validation, clinical oversight, digital-literacy support, privacy safeguards, and equitable access, and should complement rather than replace evidence-based prevention programs, direct clinical counseling, or community-based caregiver support.

Sex-based differences were also persistent. Male predominance in pTBI burden has been reported across national administrative database studies, population-based observational analyses, and mortality-surveillance reports^[^43–47^]^. These large observational studies are well suited to identifying consistent population-level patterns, but they cannot determine whether the observed differences result from biological susceptibility, injury exposure, behavioral context, injury mechanism, or differential access to care. Proposed sex-hormone and neuroprotective mechanisms are supported mainly by mechanistic and translational literature rather than by direct causal evidence from population mortality studies^[^48^]^. The present findings should therefore be interpreted as confirming a descriptive burden pattern among boys and male adolescents rather than demonstrating a biological explanation for the disparity. This pattern may help guide prevention planning for higher-risk environments while maintaining universal pediatric injury-prevention efforts.

Racial and ethnic findings require careful interpretation because CDC WONDER race reporting changed over time. For this reason, bridged-race data from 1999 to 2017 and single-race data from 2018 to 2024 were interpreted separately rather than being combined. In both periods, annual CMRs were highest among non-Hispanic American Indian or Alaska Native children at the beginning and end of the corresponding period. Similar disparities have been identified in national descriptive mortality-surveillance analyses, which provide strong population coverage but cannot explain the individual or structural mechanisms underlying the observed differences^[^44,46,49,50^]^. Potential explanatory contexts described in the literature are supported by heterogeneous observational evidence, including transportation and restraint-use surveys^[^51–53^]^, firearm-purchasing and storage surveys and descriptive injury studies^[^54–58^]^, behavioral and life-course observational studies^[^59–61^]^, and retrospective rehabilitation, socioeconomic, and insurance-based cohort analyses^[^62–66^]^. These studies identify associations between social, geographic, health-system, and injury-related factors and outcomes, but they do not establish that any single factor caused the disparities observed in the present analysis. Race and ethnicity should therefore not be interpreted as causal characteristics. Rather, these findings identify population-level disparities that may overlap with structural inequities, unequal injury exposure, geographic and trauma-system access, prevention-resource availability, socioeconomic conditions, and death-certificate reporting practices. Public-health responses should remain community-specific and equity-focused, including tribal road-safety resources, state injury-prevention programs, and violence-prevention frameworks^[^67–69^]^.

The post-2020 period requires cautious interpretation. The supporting literature includes retrospective clinical studies, ecological and policy analyses, administrative-data studies, commentaries, and official pandemic reporting rather than randomized or controlled evidence specifically evaluating pTBI mortality^[^70–74^]^. These sources describe pandemic-era changes in neurotrauma admissions, firearm-related emergency visits, family stress, interpersonal and domestic violence, child-abuse risk, and maltreatment reporting during school closures. Their heterogeneous and predominantly observational designs provide relevant temporal and social context but remain susceptible to confounding, changes in health-care utilization, reporting practices, and concurrent societal disruptions. Accordingly, they do not establish that the COVID-19 pandemic caused the later pTBI-related mortality patterns observed in this study. Pandemic-related explanations should therefore be considered contextual and hypothesis-generating rather than causal.

Geographic findings further support a systems-level interpretation. The relevant literature includes national mortality-surveillance analyses, retrospective administrative cohorts, a focused review of rural–urban pTBI disparities, prospective observational research, geospatial access analyses, and multicenter retrospective cohort studies^[^75–80^]^. National surveillance studies are well suited to identifying geographic patterns but cannot determine the patient-, community-, or health-system-level mechanisms underlying those differences^[^75^]^. The National Inpatient Sample analysis by Patel *et al* evaluated institutional pediatric trauma volume and outcomes among rural- and urban-dwelling children using retrospective administrative data, providing adjusted associational evidence that remained susceptible to coding limitations and residual confounding^[^76^]^. Similarly, the multicenter retrospective cohort study by Glass *et al* used risk adjustment to evaluate transport time, proximity to trauma care, emergency department pediatric readiness, and survival, thereby providing comparatively stronger observational evidence regarding systems of care; however, it could not establish a causal effect of transport time or pediatric readiness on an individual child’s outcome^[^79^]^. The higher crude mortality burden observed in several Mountain West, Plains, and Southern states should therefore be interpreted as a geographic burden pattern rather than evidence of a single explanatory factor. Observational evidence suggests that injury mechanism, rurality, socioeconomic conditions, trauma-system access, seat-belt policy, and firearm-storage practices may contribute to geographic variation; however, none of these factors was directly measured or causally evaluated in the present analysis^[^75,81,82^]^.

The persistent metropolitan–nonmetropolitan difference may also be interpreted within the time-sensitive “golden hour” framework of trauma care, which emphasizes rapid injury recognition, physiologic stabilization, appropriate field triage, and timely access to definitive treatment after severe injury. In pediatric neurotrauma, the golden hour should be understood as a clinical principle supporting time-sensitive care rather than as a rigid or universally validated 60-minute threshold. Observational rural–urban studies and reviews describe longer travel distances, delayed injury discovery, prolonged emergency medical services response and transport intervals, interfacility transfers, and reduced proximity to pediatric trauma and neurosurgical services as potential barriers in rural settings^[^75–80,83^]^. Delayed recognition and stabilization may prolong exposure to secondary insults such as hypoxemia, hypotension, impaired cerebral perfusion, and evolving intracranial hypertension, which can compound the initial brain injury. Current evidence-informed prehospital TBI guidelines therefore emphasize early prevention and correction of hypoxemia and hypotension, minimization of total prehospital time, and transport to facilities capable of timely neuroimaging and neurosurgical management^[^84^]^. Although these data provide biologically and clinically plausible support for the importance of timely care, they do not demonstrate that prolonged prehospital time caused the higher nonmetropolitan mortality burden observed in this study.

Several sources of residual confounding, misclassification, and information bias further limit interpretation of the geographic findings. CDC WONDER does not contain information on injury severity, Glasgow Coma Scale score, anatomic injury pattern, prehospital physiology, injury-to-discovery time, emergency medical services response or scene intervals, transport duration, interfacility transfer, trauma-center designation, neurosurgical treatment, treatment timing, socioeconomic status, insurance coverage, or local prevention policies. State and urban–rural classifications are based on the decedent’s residence and may not represent the location where the injury occurred, where emergency care was initiated, or where definitive treatment was received. Death-certificate documentation may also vary across clinicians, medical examiners, coroners, and jurisdictions, as well as over time. A pTBI may be under-recorded when another traumatic injury, external cause, or downstream complication is emphasized, while the injury mechanism and intent may be misclassified when the circumstances of death are incomplete or uncertain. Small-count suppression may additionally limit the evaluation of less common populations, mechanisms, intents, and geographic areas. Consequently, the observed geographic differences may reflect a combination of true variation in mortality burden, unequal injury exposure, differences in access to trauma and neurosurgical care, residual confounding, and variation in death investigation or coding practices. The present analysis therefore cannot establish that delayed transport, limited trauma access, or any specific policy or health-system factor caused the higher mortality burden in nonmetropolitan areas.

The place-of-death distribution adds an important but similarly descriptive care-pathway perspective. A substantial proportion of pTBI-related deaths occurred outside inpatient settings, including deaths at home, in outpatient or emergency facilities, and among children who were dead on arrival. This pattern suggests that some fatal pTBI events may occur before definitive inpatient care can alter the clinical course. However, place-of-death categories are derived from death certificates rather than from linked emergency medical services, trauma-registry, or hospital records and cannot establish injury severity, preventability, prehospital delay, treatment quality, or whether an alternative care pathway would have changed the outcome. The appropriate clinical implication is therefore not that any particular care setting caused the observed mortality differences, but that prevention and response must span the complete pathway from primary injury prevention and early recognition to field stabilization, triage, transport, transfer coordination, trauma-system readiness, and pediatric-specific neurosurgical and critical care.

## Limitations

This study has several limitations. First, the analysis used death-certificate data, which are subject to misclassification, incomplete documentation, and variation in coding practices across certifiers, medical examiners, jurisdictions, and time. A pTBI may be underreported if another traumatic injury, external cause, or downstream complication is emphasized on the death certificate, and injury circumstances may be incompletely documented. Because case identification relied on ICD-10 codes recorded on death certificates, the analysis could not validate diagnoses against clinical records, imaging findings, autopsy reports, trauma registries, emergency medical services records, or medical examiner narratives. The use of multiple-cause-of-death fields improves the capture of deaths in which pTBI contributed to mortality, but it may also include deaths in which pTBI was one of several injuries or conditions contributing to death.

Second, CDC WONDER provides aggregate mortality data and does not include individual-level clinical or contextual information needed for multivariable confounder adjustment. Important unmeasured factors include injury severity, Glasgow Coma Scale score, polytrauma, intent of injury, timing from injury to death, prehospital response time, transport distance, trauma-center designation, neurosurgical availability, intensive-care resources, comorbid conditions, socioeconomic status, insurance coverage, restraint use, firearm access or storage practices, and local injury-prevention policies. Therefore, observed differences by sex, age, race and ethnicity, geography, urban-rural status, and state should be interpreted as descriptive patterns of crude mortality burden rather than adjusted estimates of individual-level risk.

Third, all mortality rates were crude rather than age-adjusted. CMRs were used to quantify the absolute pTBI-related mortality burden within a restricted pediatric population aged 0–19 years. Age-adjusted rates were not used as the primary mortality measure because the CDC WONDER standard age-adjusted rates are not generated for the selected 5-year pediatric age groups used in this analysis. Age was therefore addressed through prespecified age-specific analyses using narrow pediatric strata. However, crude rates may still be influenced by differences in the internal age distribution of demographic and geographic subgroups, particularly when groups differ in the proportion of infants, younger children, early adolescents, and late adolescents. Therefore, subgroup comparisons should be interpreted as descriptive comparisons of crude mortality burden rather than age-standardized comparisons of mortality risk.

Fourth, findings on race and ethnicity require careful interpretation because CDC WONDER reporting changed over time. Bridged-race data were analyzed for 1999–2017, whereas single-race data were analyzed separately for 2018–2024. These categories should not be combined or interpreted as a continuous race-series across the full study period. In addition, race and ethnicity on death certificates may be misclassified, and this limitation may differentially affect some groups. Therefore, racial and ethnic findings should be interpreted as descriptive surveillance patterns rather than definitive estimates of underlying individual-level risk.

Fifth, geographic analyses have important limitations. State-level results reflect the decedent’s residence, not necessarily the location of injury or where care was received. Geographic comparisons may also be influenced by migration, regional differences in death investigation systems, coding practices, rurality, transport distance, trauma-system access, and reporting completeness. State-level results were summarized separately for 1999–2020 and 2021–2024; the 2021–2024 estimates represent a shorter contemporary interval and should be interpreted as descriptive of recent burden patterns rather than as long-term state-specific trends. Urban–rural analyses were available only through 2021 in the extracted dataset and therefore should not be interpreted as covering the entire 1999–2024 study period.

Sixth, this analysis was limited to mortality and did not capture nonfatal pTBI, emergency department visits, hospitalizations, rehabilitation outcomes, long-term disability, or quality-of-life consequences among survivors. Place-of-death categories should also be interpreted cautiously because a large proportion of deaths were classified as occurring in “other” locations, limiting more detailed interpretation of where fatal events occurred along the prehospital and hospital care pathway. In addition, injury-mechanism-specific analyses were limited to 1999–2020. Firearm-intent classifications were based on the external cause recorded as the underlying cause of death and may therefore be affected by incomplete documentation or misclassification of injury circumstances. In addition, legal-intervention estimates were predominantly suppressed because of small annual death counts, precluding reliable trend estimation for that category.

Finally, the repeated cross-sectional ecological design cannot establish causality. Temporal changes in pTBI-related mortality may have occurred alongside changes in injury exposure, prevention environments, trauma systems, emergency medical services, neurosurgical care, intensive care, death investigation, and coding practices over the study period. The perioperative management of patients undergoing cranial procedures has also continued to evolve, including growing interest in Enhanced Recovery After Surgery (ERAS) programs in cranial and spinal neurosurgery. These programs reflect a broader shift toward standardized, multimodal perioperative pathways intended to optimize postoperative recovery and reduce preventable complications^[^85^]^. However, CDC WONDER does not capture operative management, perioperative protocols, institutional ERAS implementation, postoperative complications, or treatment-level changes over time. Therefore, the present analysis cannot determine whether ERAS adoption or other advances in trauma, neurosurgical, anesthetic, or perioperative care contributed to the observed population-level mortality trends. More broadly, the present analysis cannot determine the independent effect of any specific exposure, policy, intervention, injury mechanism, or health-system factor. Accordingly, the findings should be interpreted as national surveillance estimates of crude pTBI-related mortality burden rather than as causal evidence linking specific clinical or health-system changes to mortality.

## Conclusion

In this updated 1999–2024 national analysis, pTBI-related crude mortality burden among U.S. children and adolescents declined overall, but progress was not uniform across the full study period. Mortality burden remained unevenly distributed by sex, age, race and ethnicity, and geography, with males accounting for more deaths than females; adolescents aged 15–19 years accounting for the largest number of deaths; high annual CMRs observed among non-Hispanic American Indian or Alaska Native children; and persistent state-level variation. Because rates were crude rather than age-adjusted, these findings should be interpreted as descriptive patterns of absolute mortality burden rather than age-standardized comparisons of mortality risk. Continued surveillance and targeted prevention planning are needed for pediatric populations and geographic areas with persistent burden, including intent-specific firearm-prevention strategies that distinguish homicide, suicide, and unintentional firearm discharge.

## Data Availability

The datasets generated and analyzed during the present study are publicly available on the Centers for Disease Control and Prevention's Wide-ranging Online Data for Epidemiologic Research (CDC WONDER) platform at https://wonder.cdc.gov/. All data are open-access, de-identified, and freely available for public use.
